# Mitoflash altered by metabolic stress in insulin-resistant skeletal muscle

**DOI:** 10.1007/s00109-015-1278-y

**Published:** 2015-04-25

**Authors:** Yi Ding, Huaqiang Fang, Wei Shang, Yao Xiao, Tao Sun, Ning Hou, Lin Pan, Xueting Sun, Qi Ma, Jingsong Zhou, Xianhua Wang, Xiuqin Zhang, Heping Cheng

**Affiliations:** Institute of Molecular Medicine, Peking University, Beijing, China; State Key Laboratory of Membrane Biology, Peking-Tsinghua Center for Life Sciences, Beijing, China; Department of Molecular Biophysics and Physiology, Rush University Medical Center, Chicago, IL USA

**Keywords:** Mitoflash, Mitochondrial dysfunction, Metabolic stress, Mitochondrial biomarker

## Abstract

**Abstract:**

Central to bioenergetics and reactive oxygen species (ROS) signaling, the mitochondrion plays pivotal roles in the pathogenesis of metabolic diseases. Recent advances have shown that mitochondrial flash (“mitoflash”) visualized by the biosensor mt-cpYFP affords a frequency-coded, optical readout linked to mitochondrial ROS production and energy metabolism, at the resolution of a single mitochondrion. To investigate possible mitoflash responses to metabolic stress in insulin resistance (IR), we generated an mt-cpYFP-expressing db/db mouse model with the obesity and IR phenotypes unaltered. In conjunction with in vivo imaging of skeletal muscles, we uncovered a progressive increase of mitoflash frequency along with its morphological changes. Interestingly, enhanced mitochondrial networking occurred at 12 weeks of age, and this was followed by mitochondrial fragmentation at 34 weeks. Pioglitazone treatment normalized mitoflash frequency and morphology while restored mitochondrial respiratory function and insulin sensitivity in 12 weeks mt-cpYFP db/db mice. Mechanistic study revealed that the mitoflash remodeling was associated with altered expression of proteins involved in mitochondrial dynamics and quality control. These findings indicate that mitoflash activity may serve as an optical functional readout of the mitochondria, a robust and sensitive biomarker to gauge IR stresses and their amelioration by therapeutic interventions.

**Key message:**

- In vivo detection of mitochondrial flashes in mt-cpYFP-expressing db/db mouse.
- Mitoflash frequency increased progressively with disease development.
- Mitoflash morphology revealed a biphasic change in mitochondrial networking.
- Mitoflash abnormalities and mitochondrial defects are restored by pioglitazone.
- Mitoflash may serve as a unique biomarker to gauge metabolic stress in insulin resistance.

**Electronic supplementary material:**

The online version of this article (doi:10.1007/s00109-015-1278-y) contains supplementary material, which is available to authorized users.

## Introduction

Insulin resistance (IR), a hallmark in the pathogenesis of metabolic syndrome and type 2 diabetes [1], is characterized by deterioration in glucose tolerance resulting mainly from impaired insulin-stimulated glucose metabolism in the skeletal muscle [2, 3]. Although the underlying mechanisms remain poorly understood, a strong relationship between increased intramuscular lipid content and muscle IR has been suggested [4, 5]. Diminished mitochondrial oxidative activity is thought to be responsible for the disorder of lipid metabolism and the development of IR [6–9]. Overproduction of mitochondrial reactive oxygen species (mitoROS) has been reported to elicit oxidative stress and consequently damages mitochondrial function in IR [10–12]. Interventions that scavenge mitoROS efficiently and simultaneously improve mitochondrial function and IR [10, 13].

Mitochondria are highly dynamic organelles that respond to and modulate cellular dynamics by continuously remodeling through fission, fusion, and autophagy. Emerging studies have identified mitochondrial dynamics as a novel mechanism involved in bioenergetic adaptation and mitoROS modulation. Inefficient mitochondrial respiration and exaggerated oxidative stress are characterized by a state of enhanced fission and mitigated fusion, while enhanced fusion is an important mechanism for the complementation of functional deficiencies via the sharing of soluble matrix contents [14, 15]. Defective mitochondrial dynamics has been suggested to participate in the pathogenesis of IR. It has been documented that mitochondrial fragmentation accompanied by suppressed Mitofusin 2 (Mfn2) expression occurs in obese or type 2 diabetes patients [16]. Consistently, specific Mfn2 repression is associated with decreased mitochondrial respiration and increased ROS production in skeletal muscle cells [14]. Mitophagy allows for the segregation and elimination of dysfunctional mitochondria and depends on constant fission-fusion activity [17]. Interplays between diminished mitochondrial dynamics and disturbed mitophagy have recently been indicated as causes of oxidative stress-induced declines of mitochondrial function in aging and IR [18].

Respiring mitochondria can intermittently generate mitochondrial flashes (“mitoflash”), reflecting quantal bursts of superoxide formation accompanying transient dissipation of the membrane potential and rapid depletion of the NADH along with a minor change in pH in the mitochondria [19, 20]. This novel mode of mitoROS production has been visualized by a protein biosensor, mt-cpYFP, and two ROS probes, MitoSOX and 2, 7-dichlorodihydrofluorescein diacetate [20, 21]. As a highly conserved universal mitochondrial activity, it occurs in isolated mitochondria, intact cells, ex vivo beating hearts, and living animals [19, 22–25]. While mitoflash activity is intimately interwoven with core mitochondrial functions (e.g., energy metabolism), it also constitutes a robust and sensitive responder to metabolic stress [24, 26], ischemia-reperfusion injuries, and oxidative stress [19, 27, 28]. By in vivo imaging, we have recently shown that skeletal muscle mitoflash frequency responds to changes in whole-body metabolic state [22]. These recent advances provide an opportunity for further investigating the roles of mitochondria in the pathogenesis of metabolic diseases in the integrative environment of a living animal.

In the present study, we aimed, first, to establish a method for in vivo imaging of mitoflash in an animal model of IR. Then, we designed experiments to test the hypothesis that the mitoflash serves as a biomarker of IR stress, by tracking its response to disease progression and to clinically relevant treatment. In addition, we investigated the IR-associated mitoflash changes in the context of altered mitochondrial respiration, dynamics, and mitophagic signals. The overall goal was to provide novel investigative means and new insights, from the unique viewpoint of mitoflashes, for understanding the roles of mitochondria in the pathogenesis of metabolic diseases.

## Materials and methods

### Animals

All procedures followed the principles of laboratory animal care of the National Academy of Sciences/National Research Council. db/m mice purchased from Jackson Laboratories (Bar Harbor, ME, USA) were cross mated with mt-cpYFP transgenic mice [29] to generate mt-cpYFP db/m and mt-cpYFP db/db mice. Twelve- and 34-week-old db/m or db/db male mice with and without mt-cpYFP expression were used in the following experiments for characterization of a relatively early and late stage of IR during disease development.

### Measurement of body weight, fasting blood glucose, and insulin

After 4–6 h of fasting, mouse body weight was measured; blood samples were taken from the tail vein for blood glucose measurement, and glucose levels were measured by a portable glucometer (Roche); insulin levels in serum were measured by ELISA kit (Millipore).

### Insulin tolerance test

Mice were fasted for 4 h, and insulin (Humulin® R, Eli Lilly) was injected intraperitoneally at 1 U/kg body weight. Blood samples were taken from the tail vein at indicated time points for blood glucose measurement.

### In vivo mitoflash detection by confocal microscopy

The in vivo imaging technique was developed as described previously [22]. After 4–6 h of fasting, mice were anesthetized by pentobarbital sodium (40 mg/kg body weight, i.p.), and an incision was made to expose the gastrocnemius muscle. During recording of mitoflashes, exposed muscle was immersed in isotonic balanced salt solution containing (in mM: 140 NaCl, 5 KCl, 2.5 CaCl_2_, 2 MgCl_2_, 5.6 d-glucose, and 10 HEPES, pH 7.2). Dual wavelength excitation imaging of mt-cpYFP was achieved by alternating excitation at 405 and 488 nm and detecting emission at >505 nm. Time-lapse images (*xy*-*t*) were taken at 1-s intervals.

### In vivo imaging of mitochondrial connectivity with mitochondria-targeted photoactivatable green fluorescent protein

For in vivo loading of tetramethylrhodamine methyl ester (TMRM), extracellular solution containing 500 nM TMRM was applied to the exposed muscle. Regions of interest (ROIs) were photoactivated with an intense 405-nm laser scanning beam for designated durations (typically 60 ms for an ROI of 1 × 2 μm). Multi-track scanning was performed by exciting mitochondria-targeted photoactivatable green fluorescent protein (mt-PAGFP) at 488 nm and TMRM at 543 nm, and fluorescence emission was collected at 505–530 and >560 nm, respectively.

### Image processing and mitoflash analysis

Individual mitoflash was identified with the aid of custom-devised algorithms modified from *Flashsniper* [28], and their morphological and kinetic properties, including amplitude (∆*F*/*F*_0_, maximum fluorescence increase over baseline), full duration at half maximum (FDHM), and full area at half maximum (FAHM), were measured automatically.

### Transmission electronic microscopy

Transmission electronic microscopy (TEM) images were captured on an FEI Tecnai-12 microscope at an accelerating voltage of 120 kV. A minimum of 10 micrographs from 4 to 6 mice were taken at ×12,000 or ×40,000 magnification, and the mitochondrial perimeter was measured by software Image J.

### Pioglitazone treatment

Pioglitazone (Pio) treatment was performed as previously described [30]. Briefly, Pio (Institute for Chemical Drug Control, Beijing, China) solubilized in 1.25 % hydroxypropyl methylcellulose (HPMC) was administered to 8-week-old mt-cpYFP db/db mice by oral gavage (30 mg/kg body weight, once a day) for 28 days. As a treatment control, 8-week-old mt-cpYFP db/db mice were gavaged with vehicle for 28 days.

### Measurement of mitochondrial respiratory activity

Mitochondrial respiratory activity was assessed as described previously [31]. Bioenergetic analyses of FDB myofibers were performed in an XF24 Extracellular Flux Analyzer (Seahorse Bioscience). Basal O_2_ consumption rates (pmol/min) were recorded prior to and after sequential injections of oligomycin (1 μM) and carbonyl cyanide 4-(trifluoromethoxy) phenylhydrazone (FCCP) (1 μM) to induce maximal O_2_ consumption.

### Western blot

Gastrocnemius muscle tissue was homogenized in RIPA buffer supplemented with phosphatase inhibitors (Roche) and protease inhibitors (Sigma). The following primary mouse monoclonal antibodies were used: PGC1a (Millipore), β-actin (Sigma-Aldrich), Mfn2 (Sigma-Aldrich), Optic atrophy 1 (OPA1), dynamin-related protein 1 (Drp1) (BD Transduction Laboratories), and Parkin (Cell Signaling Technology). The rabbit polyclonal antibodies Mfn1 (Santa Cruz), p62 (Cell Signaling Technology), and LC3 (Sigma-Aldrich) were also used.

### Statistical analysis

Student’s *t* test or one-way or two-way analysis of variance (ANOVA) was applied to compare differences between groups when it is appropriate. GraphPad Prism (v5, GraphPad Software, La Jolla, CA, USA) was used for statistical analysis. Differences were considered statistically significant at *p* <0.05.

## Results

### In vivo imaging of mitoflashes in insulin-resistant skeletal muscle

**Fig. 1:**
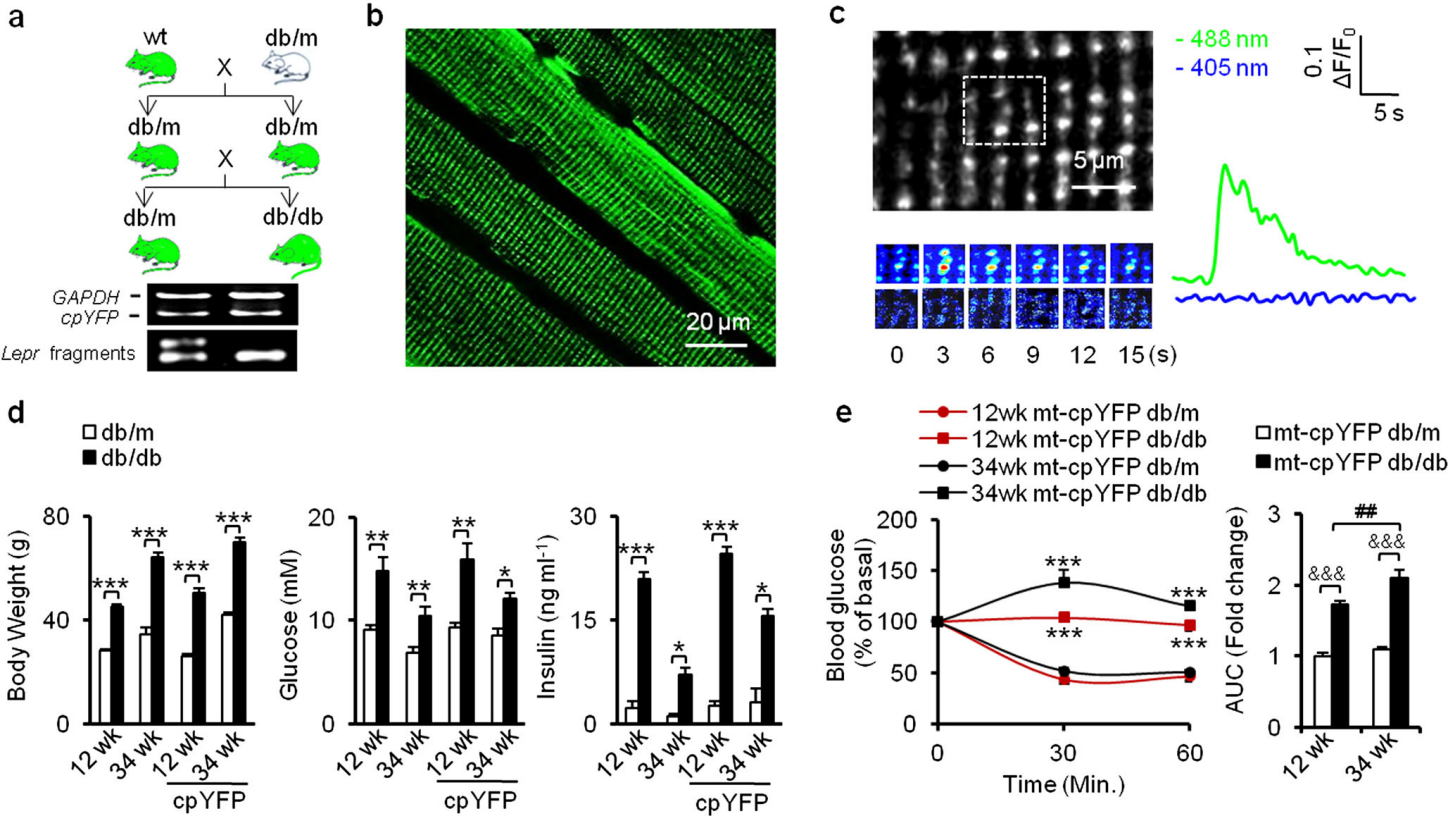
Generation of mt-cpYFP-expressing IR mouse model for in vivo imaging of skeletal muscle mitoflashes during IR progression. **a** Crossbreeding scheme for the generation of mt-cpYFP db/db mice and mt-cpYFP db/m controls. *Green*, mt-cpYFP expression. *Bottom panels*, PCR products of *mt-cpYFP* and restriction enzyme (RsaI) digestion fragments of the *Lepr* gene PCR product. **b** In vivo confocal imaging of skeletal muscle in anesthetized mice. Note the uniform expression of mt-cpYFP in different muscle fibers and in different regions within a fiber. **c** *Left panel*, a typical punctiform mitoflash from a representative db/m mouse. *Dashed box* on upper image highlights the flashing mitochondrion and its neighbors. *Lower panels*, time-lapse images of the mitoflash. *Right panel*, time courses of the mitoflash with 488- and 405-nm excitation. **d** Transgenic expression of mt-cpYFP did not alter the typical phenotypes of obesity and insulin resistance in db/db mice. Statistics of body weight (*left panel*), 4–6-h fasting blood glucose (*middle panel*), and serum insulin levels (*right panel*). **e** ITT tests showed reduced insulin sensitivity in mt-cpYFP db/db mice in both age groups (*n* = 6 mice for each group), and the area under the curve (AUC; expressed as fold change vs 12-week-old mt-cpYFP db/m) indicated progressive insulin resistance at 34 weeks in mt-cpYFP db/db mice. Data are expressed as mean ± SEM. *p < 0.05, **p < 0.01, ***p < 0.001 vs age-matched mt-cpYFP db/m mice; values were subject to Student’s *t* test. &&&*p* < 0.001 vs age-matched mt-cpYFP db/m mice, ^##^ *p* < 0.01 vs 12-week-old mt-cpYFP db/db mice; values were subject to two-way ANOVA with Tukey’s post hoc analysis

To monitor mitoflash activity in the integrative milieu of IR animals, we cross mated mt-cpYFP transgenic mice with db/m mice, both on C57BL/6 genetic background. Briefly, mt-cpYFP db/m and mt-cpYFP db/db offspring were identified by genotyping (Fig. 1a). In vivo confocal imaging was used to detect mitoflash signals in anesthetized transgenic mice. Fluorescence images of skeletal muscle confirmed the mitochondria-targeted expression of mt-cpYFP, giving rise to a striated pattern in the cells (Fig. 1b). Time-lapse images were exploited to record individual mitoflash and analyze their frequency, shape, and kinetics. A representative muscle mitoflash in an mt-cpYFP db/m mouse is shown in Fig. 1c. Typically, a mitoflash arose abruptly, peaked in 3 s, and had an FDHM of 7.65 ± 0.56 s (mean ± SEM of 51 events from 4 mice, 6–20 skeletal muscle cells/mouse). The average fractional peak increase of mt-cpYFP fluorescence (Δ*F*/*F*_0_) during a mitoflash was 0.31 ± 0.03 (mean ± SEM of 51 events from 4 mice, 6–20 skeletal muscle cells/mouse), in agreement with previous reports [22].

Similar to the db/db mice, mt-cpYFP db/db mice displayed greater body weight, elevated blood glucose, and higher insulin levels when compared with their mt-cpYFP db/m littermates (Fig. 1d). Insulin tolerance tests (ITTs) revealed that whole-body insulin sensitivity was clearly reduced at 12 weeks and was further blunted at 34 weeks in mt-cpYFP db/db mice (Fig. 1e). Thus, the transgenic expression of mt-cpYFP did not alter the manifestation of obesity, glucose intolerance, and IR progression in the db/db mice [32], validating the mt-cpYFP db/db mouse as an IR model. These results laid the foundation for the use of mitoflashes in the investigation of possible disease-related mitochondrial dysfunction in living animals.

### Progressive increase of mitoflash frequency in mt-cpYFP db/db mice

**Fig. 2:**
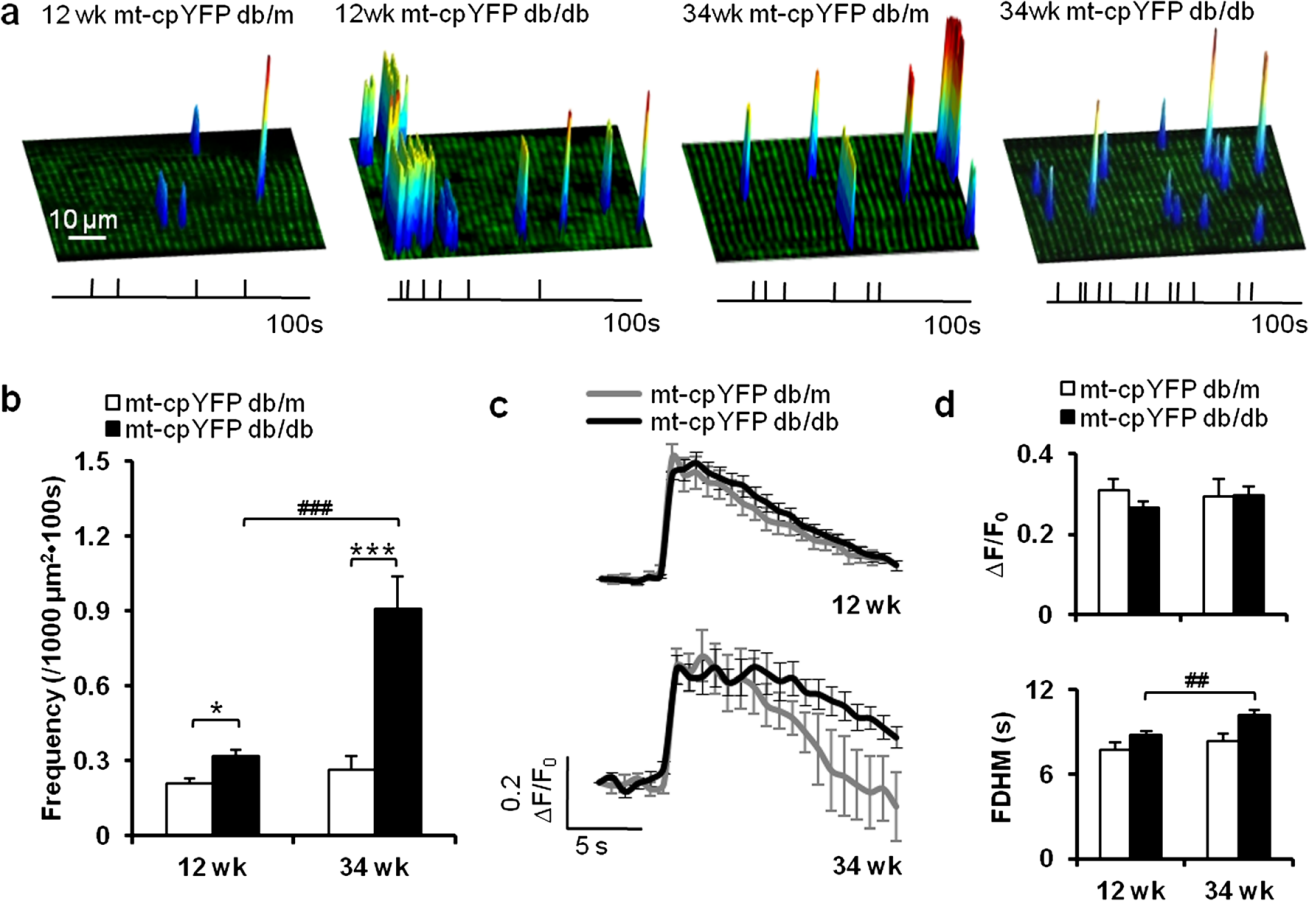
Altered frequency and unitary properties of mitoflashes in IR skeletal muscle. **a** Surface plots of the frequency, amplitude, location, and spatial properties of mitoflashes in representative skeletal muscles from 12- and 34-week-old mt-cpYFP db/m and mt-cpYFP db/db mice. *Vertical ticks beneath the images* mark the timing of these events during a 100-s acquisition window. **b** Mitoflash frequency increased during disease progression in mt-cpYFP db/db mice. **c**, **d** Ensemble-averaged time courses of mitoflashes (**c**) and quantitation of mitoflash amplitude (∆*F*/*F*_0_) and kinetics (full duration at half maximum, FDHM) (**d**). Data are expressed as mean ± SEM. *p < 0.05, ***p < 0.001 vs age-matched mt-cpYFP db/m mice; ^##^ *p* < 0.01, ^###^ *p* < 0.001 vs 12-week-old mt-cpYFP db/db mice; values were subject to two-way ANOVA with Tukey’s post hoc analysis

To determine whether and how mitoflash changes during the progression of IR, we acquired and characterized mitoflash events from mt-cpYFP db/m and mt-cpYFP db/db mice at 12 and 34 weeks of age (Fig. 2a). At 12 weeks, the rate of occurrence of mitoflashes showed a marginal but significant increase in mt-cpYFP db/db mice, from 0.21 ± 0.02/1000 μm^2^ · 100 s in mt-cpYFP db/m control mice (*n* = 173 files from 4 mice, 6–20 skeletal muscle cells/mouse) to 0.32 ± 0.03/1000 μm^2^ · 100 s (*n* = 240 files from 7 mice, 7–25 skeletal muscle cells/mouse), accompanied by overt obesity, hyperglycemia, hyperinsulinemia, and IR (Fig. 1d, e). At 34 weeks, the mitoflash frequency in mt-cpYFP db/db mice was significantly increased compared to that at 12 weeks and was 243 % higher than that in the age-matched mt-cpYFP db/m control group (Fig. 2b).

For quantitative analysis of the properties of individual mitoflash, we performed ensemble averaging of their time course and parametric measurement of their morphology (Fig. 2c, d). At 12 weeks, the mitoflash from IR and control animals were virtually identical in terms of amplitude and time course. At 34 weeks, the amplitude remained unchanged while the kinetics of decay was moderately retarded (Fig. 2d), consistent with the notion that changes of mitoflash activity occur mainly in a frequency-modulated manner. Collectively, these in vivo data showed that mitoflash activity is mildly altered in the early phase of IR, but overt dysregulation develops during disease progression.

### Altered mitochondrial functional network reflected by mitoflash morphology in mt-cpYFP db/db mice

Apart from punctiform events each confined to a round or elliptic mitochondrion, arrays of neighboring mitochondria along the same or a few neighboring *Z*-lines can ignite and extinguish synchronously, giving rise to either a linear (length >2 μm) or a lamellar (≥2 sarcomeres) appearance of the mitoflash [22, 33]. These communal events might reflect physical interconnectivity among neighboring mitochondria, a notion supported by data from electron microscopy [34]. As such, mitoflash morphology affords a novel means to assess local mitochondrial networking.

**Fig. 3:**
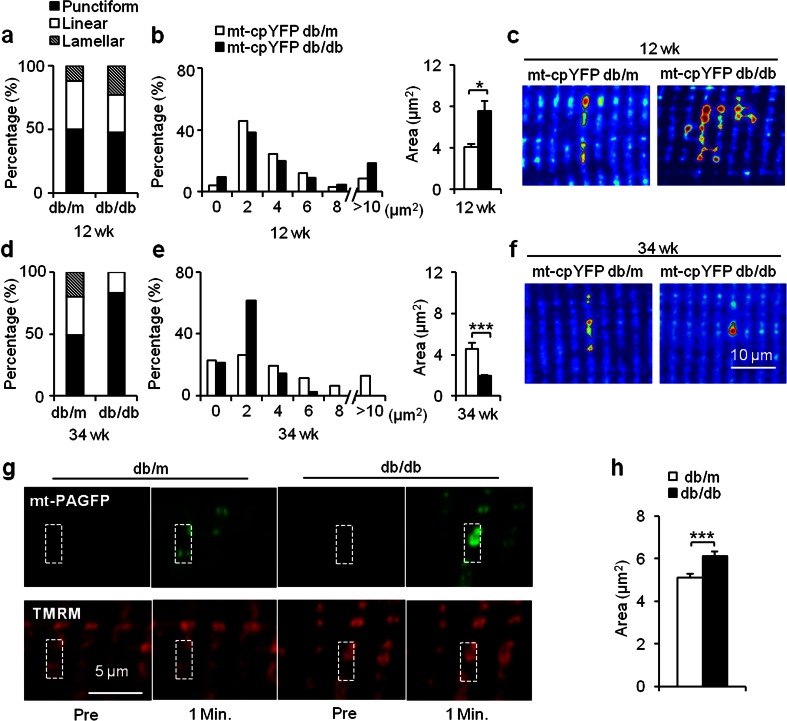
Biphasic changes of spatial properties of mitoflashes during IR progression. **a–f** Using an automated mitoflash detection algorithm, the proportions of punctiform, linear (length >2 μm), and lamellar (≥2 sarcomeres) mitoflashes were quantified (**a**, **d**), and mitoflash areas were measured in different groups (**b**, **e**). Note that average mitoflash size was enlarged at 12 weeks (*upper panel*) and diminished at 34 weeks in mt-cpYFP db/db group (*lower panel*). Representative images show mitoflashes of punctiform (*bottom right*), linear (*top left and bottom left*), and lamellar shapes (*top right*). Contours delineating mitoflash boundaries were generated automatically by the flash detection algorithm (**c**, **f**). **g** Hyper-connectivity of local mitochondrial networking in early IR was confirmed by the spatial spreading of photoactivated mt-PAGFP (*upper images*). A pair of images of the same region was taken immediately before (Pre) and 1 min after photoactivation. Note that mt-PAGFP signals co-localized with TMRM and spread beyond the region of photoactivation. **h** Average area of photoactivated mt-PAGFP was increased in skeletal muscle of 12-week-old db/db mice compared to that in db/m mice. Data are expressed as mean ± SEM. *p < 0.05, **p < 0.01, ***p < 0.001 vs age-matched mt-cpYFP db/m or db/m mice; values were subject to Student’s *t* test

Using an automated mitoflash detection algorithm [28], we classified mitoflashes into three categories—punctiform, linear, and lamellar—and measured the area. At 12 weeks, more lamellar mitoflashes were detected in mt-cpYFP db/db, and the histogram of the mitoflash area was shifted rightward, with network mitoflash events (>10 μm^2^) twice as frequent as in the mt-cpYFP db/m group (Fig. 3a, b). The average area of a mitoflash increased from 4.08 μm^2^ (*n* = 90 events from 4 mice, 6–20 skeletal muscle cells/mouse, average observation area = 3823.1 ± 167.9 μm^2^/cell) in mt-cpYFP db/m mice to 7.60 μm^2^ in mt-cpYFP db/db mice (*n* = 622 events from 7 mice, 7–25 skeletal muscle cells/mouse, average observation area = 3848.9 ± 134.1 μm^2^/cell, *p* < 0.05 vs control) (Fig. 3c). This finding showed enhanced local mitoflash networking is a prominent feature of the mitochondrial response in skeletal muscles in the relatively early phase of IR.

As the IR progressed and the mitoflash frequency increased (Figs. 1 and 2), there was a marked reduction in the proportion of linear mitoflashes and a total absence of lamellar mitoflashes in mt-cpYFP db/db mice at 34 weeks (Fig. 3d–f). The distribution of mitoflash size displayed a leftward shift with a prominent mode at ~2 μm^2^, and the average area diminished from 4.53 μm^2^ in mt-cpYFP db/m mice (*n* = 42 events from 4 mice, 7–14 skeletal muscle cells/mouse, average observation area = 3192.5 ± 70.9 μm^2^/cell) to 1.92 μm^2^ in mt-cpYFP db/db mice (*n* = 158 events from 4 mice, 7–13 skeletal muscle cells/mouse, average observation area = 3176.7 ± 56.7 μm^2^/cell, *p* < 0.001 vs control) (Fig. 3d–f). Thus, after initial enhancement of local mitochondrial networking, sustained IR stress causes fragmentation such that small but more frequent punctiform mitoflashes dominate.

That IR stress promotes mitochondrial fission and fragmentation is in general agreement with reports by several groups [14, 16, 35]. However, that IR stress initially promotes local mitochondrial networking, suggestive of enhanced physical and functional communication among mitochondria, came as a surprise. To gain independent support, we used mt-PAGFP, which has been frequently used to reveal mitochondrial interconnection changes [36]. In mt-PAGFP-expressing skeletal muscle, we detected a 40-fold increase in local mt-PAGFP fluorescence upon 60-ms illumination with a 405-nm laser, while the TMRM staining, which co-localized with photoactivated mt-PAGFP and reflects the mitochondrial membrane potential [36], remained unchanged. Notably, not all photoactivated mt-PAGFP signals were confined to sites of laser illumination (Fig. 3g); rather, it spread along the *Z*-line and even across two or more sarcomeres. The average area of the mt-PAGFP signal was 5.06 ± 0.17 μm^2^ (*n* = 108 files from 4 mice, 5–7 skeletal muscle cells/mouse, average observation area = 3255.7 ± 135.7 μm^2^/cell) in db/m mice and was markedly increased (6.18 ± 0.20 μm^2^, *n* = 176 files from 4 mice, 5–8 skeletal muscle cells/mouse, average observation area = 3794.6 ± 245.8 μm^2^/cell, *p* < 0.001 vs control) in db/db mice (Fig. 3h), substantiating the notion that local mitochondrial networking is enhanced in skeletal muscle in the relatively early phase of IR.

### Biphasic mitochondrial morphological change during the progression of insulin resistance

**Fig. 4:**
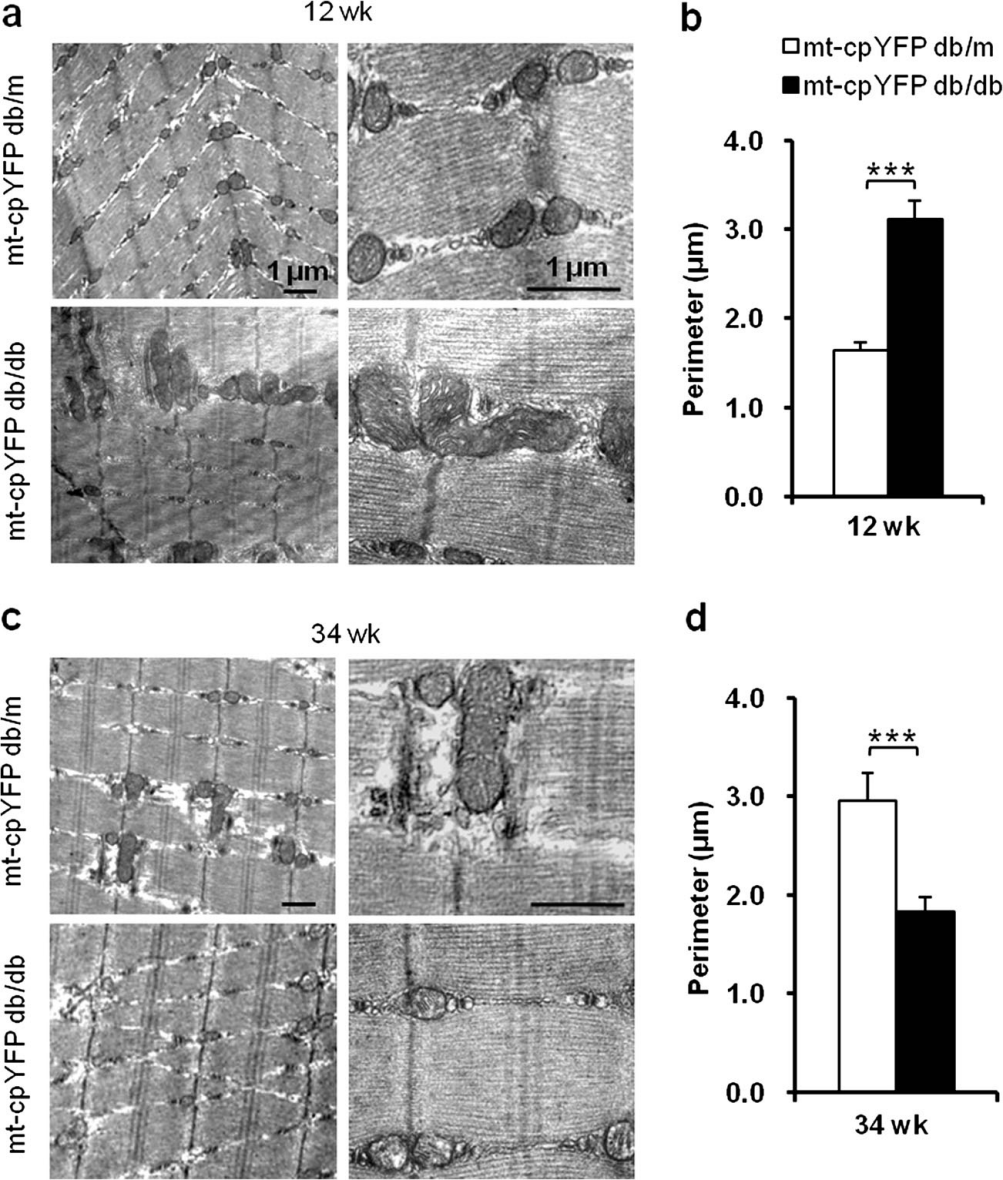
TEM micrographs of mitochondrial networking changes in IR skeletal muscle. Representative TEM micrographs in (**a**) and (**c**) show altered mitochondrial morphology and networking in skeletal muscles of 12- and 34-week-old mt-cpYFP db/db mice compared to mt-cpYFP db/m littermates. *Left*, ×12,000; *right*, ×40,000. Statistics of perimeters of mitochondria in TEM micrographs in (**b**) and (**d**) show enlargement and enhanced connectivity at 12 weeks followed by fragmentation at 34 weeks (*n* = 4 mice for each group). Data are expressed as mean ± SEM. ***p < 0.001 vs age-matched mt-cpYFP db/m mice; values were subject to Student’s *t* test

To determine the structural basis underlying this biphasic change of mitoflash morphology during disease development, we next used TEM to complement the optical fluorescent imaging approach. In control mice at 12 weeks, most mitochondria were spheroid or bean shaped and appeared at the dyads of *Z*-lines. A small proportion of elongated tubular mitochondria were also observed. In mt-cpYFP db/db mice, congruent with increased communal mitoflash activity and area, more elongated tubular and irregularly shaped mitochondria with an increased perimeter were seen (Fig. 4a, b).

In comparison, elongated mitochondria were found at a slightly higher frequency in muscle from 34-week-old mt-cpYFP db/m mice. However, elongated, interconnected, tubular mitochondria were rarely observed in 34-week-old mt-cpYFP db/db mice. Instead, most of the mitochondria were smaller, and the averaged mitochondrial perimeter seen in TEM micrographs was significantly decreased (Fig. 4c, d). In conjunction with confocal imaging of mitoflash shape and local mt-PAGFP diffusion, these finding revealed a biphasic change in mitochondrial networking at different stages of disease in the IR mouse model.

### Altered expression of proteins for mitochondrial dynamics and quality control during the progression of insulin resistance

**Fig. 5:**
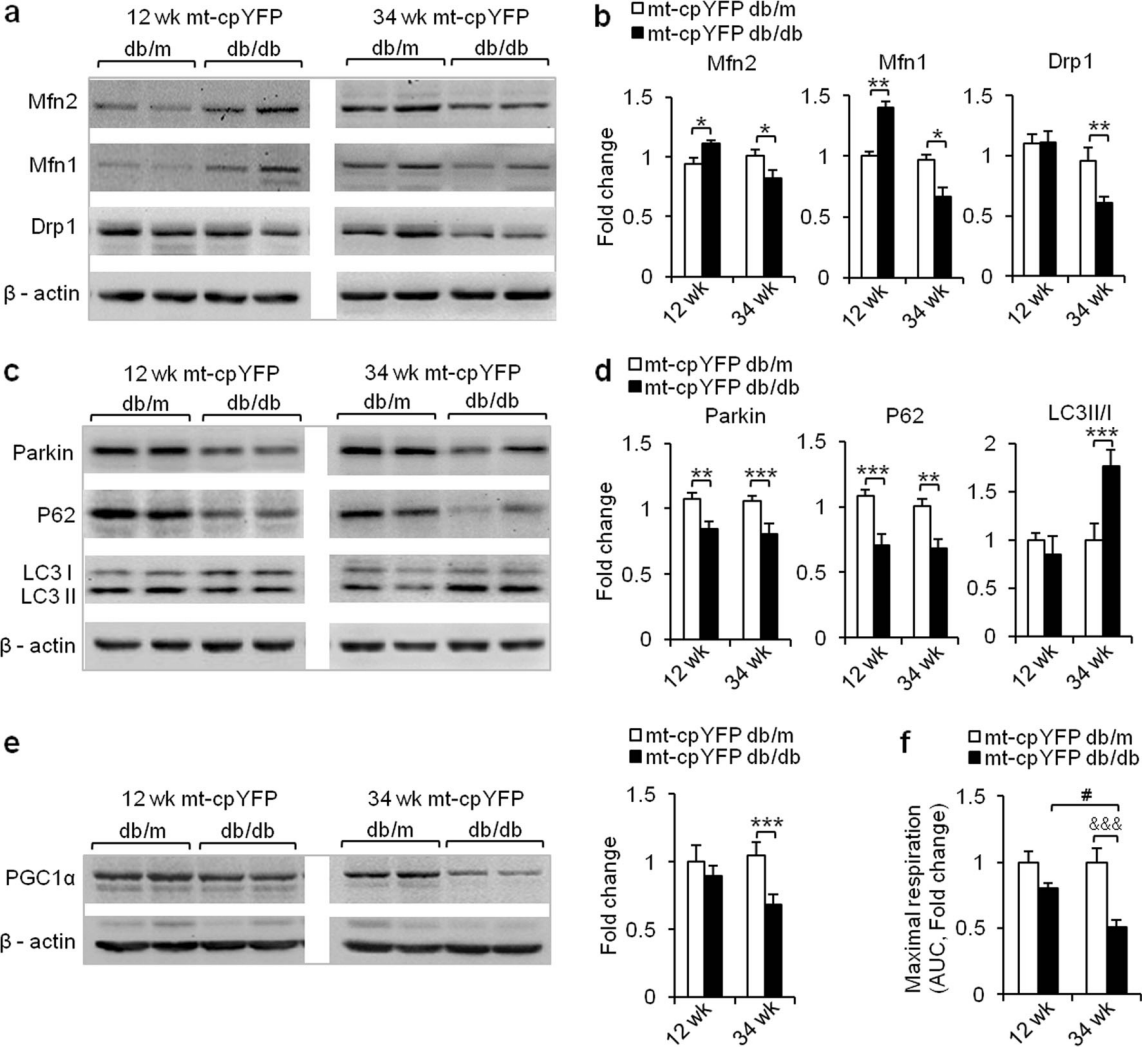
Altered expression of proteins for mitochondrial dynamics and mitophagy, and deficient mitochondrial respiration in IR skeletal muscle. **a–d** Representative Western blots (**a**, **c**) and statistics (**b**, **d**) show that the expression of essential proteins involved in mitochondrial dynamics and quality control changed during IR progression; values were recorded as fold change vs age-matched mt-cpYFP db/m littermates (*n* = 4–6 mice). **e** Representative Western blots and statistics show that the expression of master regulator of mitochondrial oxidative phosphorylation, PGC1α. Values were recorded as fold change vs age-matched mt-cpYFP db/m littermates (*n* = 4–6 mice). **f** Using the XF24 Extracellular Flux Analyzer (Seahorse Bioscience), isolated FDB muscles from each group were sequentially treated with oligomycin (1 μM) and FCCP (1 μM) to assess the maximal respiration capacity of mitochondria. Area under curves of O_2_ consumption rates were calculated for comparison among groups. Values were recorded as fold change vs 12-week-old mt-cpYFP db/m littermates (*n* = 4 mice for each group). Data are expressed as mean ± SEM. *p < 0.05, **p < 0.01, ***p < 0.001 vs age-matched mt-cpYFP db/m mice; values were subject to Student’s *t* test. &&&*p* < 0.001 vs age-matched mt-cpYFP db/m, ^#^ *p* < 0.05 vs 12-week-old mt-cpYFP db/db; values were subject to two-way ANOVA with Tukey’s post hoc analysis

We next investigated the possible molecular basis of these morphologic changes at different stages in the development of IR. We found that the levels of Mfn2 and Mfn1, which promotes mitochondrial fusion, were significantly increased at 12 weeks, coincident with enhanced mitochondrial networking and enlargement at that time. However, these upregulations disappeared at 34 weeks, when fragmentation of mitochondrial network was evident. Pro-fission protein Drp1 only manifested a significant reduction at 34 weeks (Fig. 5a, b).

It has been shown that mitochondrial dynamics also contribute to mitochondrial quality control through mitophagy, and inhibition of either Mfn2 or Drp1 leads to deficient turnover and dysfunction [37, 38]. During mitophagy, cytosolic Parkin targets and translocates to depolarized mitochondria [39]. The autophagy adaptor p62 is recruited [40] and further binds to microtubule-associated protein-1 light chain II (LC3 II), which is the autophagosome-associated form of LC3. Subsequently, LC3 II entrapped inside autophagosomes is degraded [41]. Investigation of molecules involved in the mitophagy pathway revealed that p62 and Parkin protein levels were decreased in mt-cpYFP db/db mice. Meanwhile, a significant accumulation of LC3II was found in these mice at 34 weeks, suggesting an accumulation of autolysosomes. These results indicated that mitophagy is markedly retarded in mt-cpYFP db/db mice at 34 weeks (Fig. 5c, d). Concomitant with this, mitochondrial respiratory function was diminished as evidenced by the reduced expression of PGC1α, which largely controls mitochondrial oxidative phosphorylation [42], and maximal respiratory capacity in mt-cpYFP db/db mice (Fig. 5e, f), along with the reduction of mitoflash area and exaggerated mitochondrial networking fragmentation. Taking together the data from molecular profiling, ultrastructural analysis, and mitoflash measurement, we conclude that IR stress causes characteristic compensatory responses in the mitochondria at 12 weeks that proceed to a decompensated state at 34 weeks in mt-cpYFP db/db mice.

### Reversal of altered mitoflash activity and respiratory function in pioglitazone-treated mt-cpYFP db/db mice

The data thus far suggested that mitoflash activity may serve as a sensitive and robust biomarker of IR stress and disease progression. In particular, mitoflash appeared to reflect both the metabolic status and the local networking of mitochondria. To test this hypothesis, we examined the mitochondrial responses to clinically relevant treatment of IR.

**Fig. 6:**
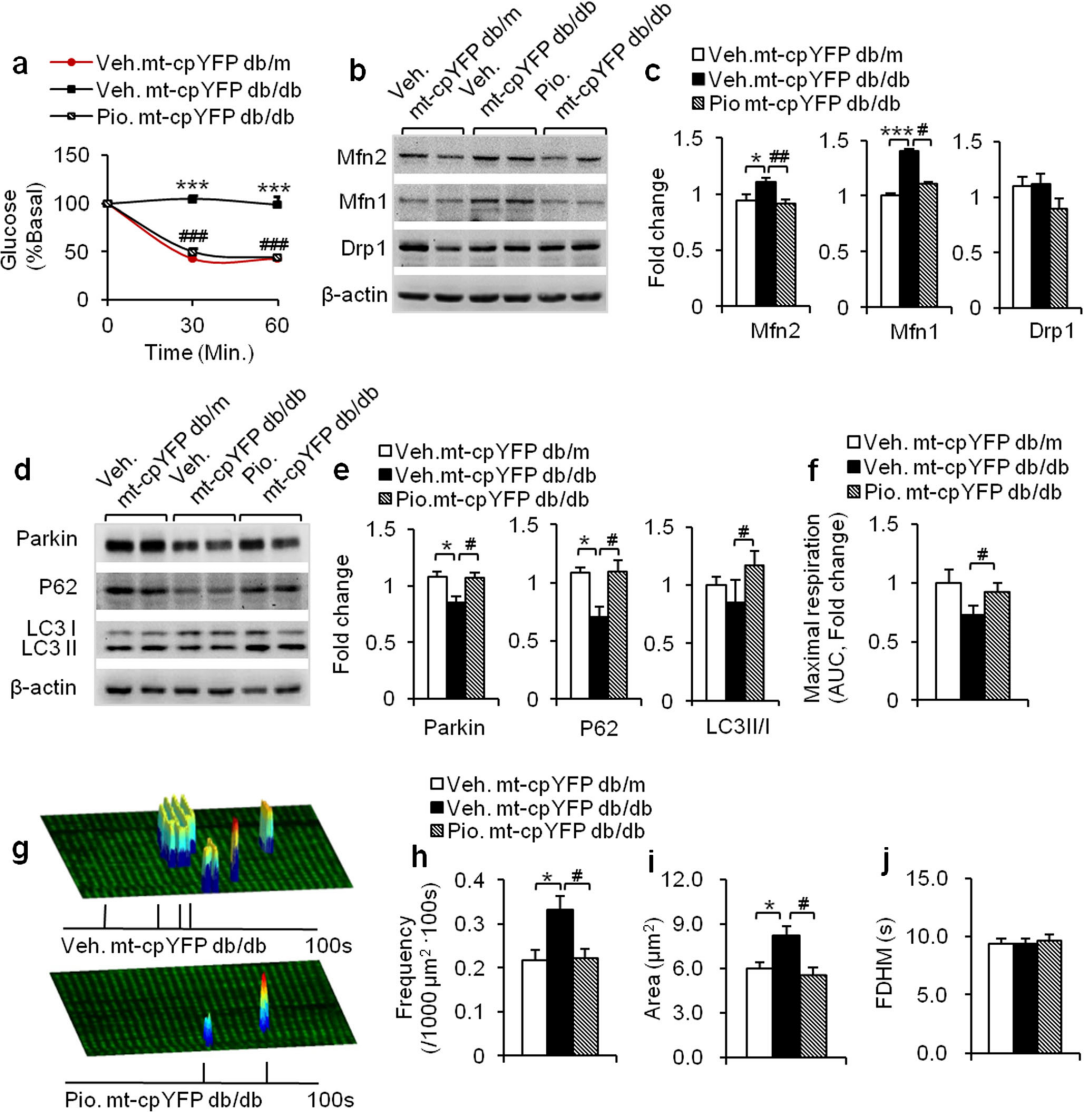
Pioglitazone treatment normalizes mitochondrial structure, respiratory function, and mitoflash activity while restoring insulin sensitivity. **a** In 12-week-old mt-cpYFP db/db mice, systemic insulin sensitivity was restored by pioglitazone (Pio) as indicated by the insulin tolerance test in vehicle (Veh)- and Pio-treated mt-cpYFP db/db mice (*n* = 6 mice for each group). **b–e** Pio effects on expression of Mfn2, Mfn1, Drp1, Parkin, p62, and LC3 in skeletal muscle; values were recorded as fold change vs Veh-treated mt-cpYFP db/m littermates (*n* = 4 mice for each group). **f** Maximal mitochondrial respiration was significantly improved. Area under curves of O_2_ consumption rates were calculated for comparison among groups. Values were recorded as fold change vs Veh-treated mt-cpYFP db/m littermates (*n* = 4 mice for each group). **g-j** Typical images (**g**) and statistics of Pio effects on mitoflash frequency (**h**), spatial area (**i**), and FDHM (**j**) (*n* = 4–8 mice for each group). Data are expressed as mean ± SEM. *p < 0.05, ***p < 0.001 vs Veh-treated db/m mice, ^#^ *p* < 0.05, ^##^ *p*<0.01, ^###^ *p*<0.001 vs Veh-treated db/db mice; values were subject to one-way ANOVA with Tukey’s post hoc analysis

Pioglitazone (Pio) is one of the thiazolidinediones (TZDs) that are commonly used to treat metabolic syndrome and type 2 diabetes in clinical practice [43]. A previous study has shown that rosiglitazone, another TZDs, upregulates the expression of genes involved in mitochondrial biogenesis and changes the mitochondrial structure in the adipose tissue of db/db mice [44, 45]. In obese IR mice, Pio ameliorates IR in the skeletal muscle [30]. In 12-week-old mt-cpYFP db/db mice, ITT confirmed the efficacy of Pio in restoring whole-body insulin sensitivity (Fig. 6a). Molecular profiling revealed that Mfn2 and Mfn1 were downregulated, and Drp1 was unchanged, all these dynamics-related proteins returning to or remaining at control levels (Fig. 6b, c). In terms of mitophagy-related signaling, the expression of Parkin, p62, and LC3II were also normalized (Fig. 6d, e). Functionally, the maximal mitochondrial respiration was increased by 26.4 % after Pio treatment compared to the vehicle-treated group (Fig. 6f). These results suggested that Pio treatment ameliorates the alterations in mitochondrial function, dynamics, and morphology, in good agreement with the notion that TZDs help to preserve mitochondrial function and structure in IR.

More importantly, we found that Pio treatment significantly decreased mitoflash frequency by 33.3 %, to a level comparable to that in mt-cpYFP db/m mice (Fig. 6g, h). Furthermore, the increases in mitoflash size and FDHM associated with early IR were also initially reversed after Pio treatment (Fig. 6i, j), indicating the restoration of mitochondrial metabolism and local networking. Thus, the changes in mitoflash activity and unitary properties as well as changes in mitochondrial functions, dynamics, and the quality-control machinery were essentially normalized with the reversal of IR and associated stresses. In addition, these results strongly support the notion that mitoflash can be exploited as a novel biomarker to gauge metabolic disease progression and therapeutic efficacy in living animals.

## Discussion

In this study, we have characterized, for the first time, mitoflash changes in the IR mt-cpYFP transgenic mouse model. We dissected the alterations of mitoflash characteristics with IR development and documented the mitoflash response to therapeutic intervention. The main finding was that mitoflash can serves as a unique biomarker of disease-associated stress in the db/db mouse model of IR. Specifically, mitoflash frequency was mildly increased early in IR at 12 weeks and was overtly elevated at 34 weeks. This progressive increase in mitoflash frequency occurred in parallel with the impairment of mitochondrial respiration and deterioration of mitophagic signaling. The latter was indexed by sustained decreases of Parkin and p62 expression and an increase of LC3II accumulation, indicating repressed mitophagic flux. More importantly, we found that treatment of Pio, one of the commonly used TZDs, effectively reversed the mitoflash responses, while restoring insulin sensitivity in the relatively early stage of IR. Pio treatment also simultaneously normalized mitochondrial respiration, local mitochondrial networking, and the expression of major proteins mediating mitochondrial dynamics and quality control. Taken together, these lines of evidence strongly support our hypothesis that the mitoflash is a biomarker which gauges the state of disease progression as well as the efficacy of therapeutic intervention.

The above findings fit well with the prevalent view that the mitochondrion is central to the development of many types of metabolic diseases. Cumulative metabolic stress has been suggested to be involved in oxidative stress-induced IR. As the main site of cellular energy metabolism, the mitochondrion is the first-line sensor of and defender against metabolic stressors. Moreover, metabolic stress is often interlinked with oxidative stress—increased access to lipid, glucose, and insulin is known to exacerbate oxidative stress [46, 47]. This is because, in the process of energy metabolism, the mitochondria generate superoxide anions as the precursor of other types of ROS and also enzymes for antioxidant defense. Hence, mitochondria are also the first-line sensor of and defender against diverse oxidative stresses. Indeed, previous in vitro and in vivo experiments have shown that glucose and insulin acutely stimulate mitoflash activity in the skeletal muscle [19, 46] and basal ROS production also contributes to mitoflash ignition [26]. The present results indicate that the mitoflash machinery is also a point of convergence for IR-associated metabolic and oxidative stresses, and optical measurement of mitoflash affords a novel means for the investigation of the mitochondrial stress response in living animals.

The monophasic increase of mitoflash frequency during IR progression was in sharp contrast to the biphasic change of local mitochondrial networking, as revealed by mitoflash area, the spread of mt-PAGFP, and TEM data. The latter was manifested as hyperconnectivity and enlargement of mitochondria at 12 weeks and eventual mitochondrial networking fragmentation at 34 weeks. Functionally, mitochondrial dynamics exert a significant impact on adaptation to altered metabolic states. Mitochondrial fusion helps to maximize the fidelity of oxidative respiration by complementation among mitochondria [15, 48], and fragmentation by fission or disconnection severely compromises it. In this scenario, we propose that transiently enhanced local mitochondrial networking at 12 weeks constitutes a compensatory mechanism, whereas eventual mitochondrial networking fragmentation at 34 weeks reflects a detrimental consequence of the IR stress, which may drive disease progression in a vicious cycle. Similar to our observation in 34-week-old mt-cpYFP db/db mice, it is reported that mitochondrial dynamics of skeletal muscle was impaired in long-term HFD (40 weeks) induced obese mice [49]. This interpretation is further supported by the finding that Pio treatment, while normalizing insulin sensitivity, effectively reversed the change in local mitochondrial networking.

Mechanistically, we provide evidence that major mediators in the mitochondrial fusion-fission machinery may be responsible for the biphasic changes of local mitochondrial networks in IR db/db mice. In particular, there were similar biphasic changes in Mfn2 and Mfn1, which promotes mitochondrial fusion, increasing at 12 weeks followed by a decrease at 34 weeks. The result is consistent with the mitochondrial networking fragmentation and decreased Mfn2 expression in muscles from patients with type 2 diabetes and obese humans [16]. However, it is opposite to the prediction from reduced Drp1 expression at 34 weeks, which would repress mitochondrial fission and fragmentation. At this time point, the suppressive effects of Mfn2 appear to be dominant in the interplay among different mediators of mitochondrial dynamics.

By in vivo imaging skeletal muscle in a newly established biosensor-expressing db/db mouse model, we demonstrated a progressive increase of mitoflash frequency during IR and its reversal by Pio treatment in relatively early IR. We also uncovered that, prior to networking fragmentation, there was a transient, Pio-reversible enhancement of local mitochondrial networking in early IR, arising from changes in mediators of mitochondrial dynamics and quality control. These findings support a concept that mitoflash can be used as the optical readout of mitochondrial function and its stress response, gaining unique insights into free radical formation and energy metabolism as well as mitochondrial dynamics, radical formation, energy metabolism as well as mitochondrial dynamics. In addition, the in vivo imaging methods allow for multiple observations in single animals, which enhance the resolution of mitochondrial functional changes during disease progression. Collectively, our findings support a central role of the mitochondria in the pathogenesis of metabolic disease and suggest that mitoflash may serve as a unique biomarker to gauge IR stresses and their amelioration by therapeutic interventions.

## Electronic supplementary material

ESM 1 (PDF 24 kb)
