# Supplementary material for: Mitoflash altered by metabolic stress in insulin-resistant skeletal muscle
Source: J Mol Med (Berl). 2015 Apr 25;93(10):1119–30. doi: 10.1007/s00109-015-1278-y (PMC4589561; doi:10.1007/s00109-015-1278-y)
Supplement: Supplementary file 1 — (PDF 24 kb) [file 109_2015_1278_MOESM1_ESM.pdf]

J Mol Med 2015

Electronic Supplementary Material

Mitoflash altered by metabolic stress in insulin-resistant skeletal muscle

Yi Ding, Huaqiang Fang, Wei Shang, Yao Xiao, Tao Sun, Ning Hou, Lin Pan, Xueting Sun, Qi Ma, Jingsong Zhou, Xianhua Wang, Xiuqin Zhang, and Heping Cheng

Supplemental methods

Genotyping of mt-cpYFP db/db mice Genotyping for *mt-cpYFP* insertion was performed by PCR using mouse-tail DNA that isolated with a genomic DNA isolation kit (Qiagen). PCR primers for *mt-cpYFP*: 5’-GCACAAGCTGGAGTACAACGGT-3’ (forward) and 5’-GATGACCTCTTTATAGCCAACCT-3’ (reverse). *Gapdh* fragment was used as internal control (forward primer: 5’-AGGCCGGTGCTGAGTATGTC-3’ and reverse primer: 5’-TGCCTGCTTCACCACCTTCT-3’). Specific primers were used for production of *Lepr* fragment (forward primer: 5’- AGA ACG GAC ACT CTT TGA AGT CTC-3’ and reverse primer: 5’- CAT TCA AAC CAT AGT TTA GGT TTG TGT-3’). The *Leptin receptor* (*Lepr*) mutation was detected by restriction enzyme (RsaI) digested fragments of the *Lepr* gene PCR product.

In vivo electroporation mt-PAGFP was transiently expressed in muscles of 12-week-old mice by in vivo electroporation. In vivo electroporation method was

adapted from a previous report [1]. Briefly, in the hindlimb of anaesthetized 12-week-old db/m or db/db mice (pentobarbital sodium, 40 mg/kg body weight, i.p.), hyaluronidase (Sigma) and mt-PAGFP plasmid (a kind gift from Dr. Mariusz Karbowski) were injected subcutaneously in sequence. Electronic stimulation with 20 pulses of square-wave were applied at 1 Hz (ECM 830 Electro Square Porator, BTX). In vivo imaging was carried out 10 days later using procedures similar to those described above.

Single skeletal muscle cell isolation and culture The method was adapted from previous report [2]. Briefly, surgically-excised mouse flexor digitorum brevis (FDB) muscle was incubated in DMEM supplemented with 2% FBS and 4 mg/ml collagenase A for 1.5-2 h to yield single FDB myofibers. Aliquots were taken randomly and deposited into each well of XF24 cell culture microplates and cultured for 12-16 h.

Reference

1. Luo G, Yi J, Ma C, Xiao Y, Yi F, Yu T, Zhou J (2013) Defective mitochondrial dynamics is an early event in skeletal muscle of an amyotrophic lateral sclerosis mouse model. PLoS One 8: e82112.
2. Schuh RA, Jackson KC, Khairallah RJ, Ward CW, Spangenburg EE (2012) Measuring mitochondrial respiration in intact single muscle fibers. Am J Physiol Regul Integr Comp Physiol 302: R712-719.
